# The American Institute of Homœopathy

**Published:** 1887-04

**Authors:** Ad. Lippe

**Affiliations:** Philadelphia


### THE AMERICAN INSTITUTE OF HOMŒOPATHY.

AD. LIPPE, M. D., PHILADELPHIA.

In the *Transactions of the Thirty-ninth Session of the American Institute of Homœopathy* (1886), page 45, will be found the report of Chas. Mohr, M. D., Chairman of a committee appointed in 1882 on "Homœopathy in the *Encyclopedia Americana*." It is here reprinted. At the meeting of 1881 Drs. C. Mohr, J. C. Morgan, and J. C. Guernsey were appointed a committee to have the subject "Homœopathy" properly treated in the reprint edition of the *Encyclopedia Britannica*, gross injustice having been done the homœopathic school of medicine in the original Edinburgh edition.

At the meeting in 1882 this Committee reported having conferred with the publishers, who answered that the article in the reprint edition must read as it does in the original, and that T. M. Stoddart & Co. had agreed to represent Homœopathy properly in an article in the American supplement. The Committee recommended that a duly qualified committee be appointed to examine said article, with authority to approve of its publication if found satisfactory. On motion, the report was received and the Committee continued, Dr. Pemberton Dudley being added. In 1883 the enlarged Committee reported progress:

"Your Committee now take great pleasure in reporting that the Messrs. Hubbard, who are the publishers of the American supplement of the *Encyclopedia Britannica*, styled the *Encyclopedia Americana*, secured the services of Dr. Horace Howard Furness, the well-known Shakespearian scholar, to write an article on 'Homœopathy;' that this article was duly submitted to your Committee, and, on their approval, has been published in the third volume of said *Encyclopedia Americana*, just issued. A copy of said article is herewith appended, and your Committee believe that it embraces all the information that an encyclopedia article on this subject can give.

"Dr. Furness merits the thanks of every homœopathist for the able manner in which he has treated his great subject.

"Respectfully submitted,

"C. Mohr, M. D.,
"J. C. Morgan, M. D.,
"J. C. Guernsey, M. D.,
"P. Dudley, M. D.

"On motion, the report was accepted and referred, and a vote of thanks extended to Dr. H. H. Furness, of Wallingford, Pa., for his able article."

It is very gratifying to find that this Committee and the American Institute *approve* of the most excellent paper on Homœopathy written by H. H. Furness, Esq., and have honored him with a vote of thanks.

Various articles on Hahnemann and on Homœopathy had formerly been published in various encyclopedias. These articles were written by professional and *interested* parties—by allopathists and pseudo-homœopaths. After due deliberation, some old members of the profession, desiring that Homœopathy should be fairly presented before an intelligent public, became convinced that such a paper should be written by a ripe scholar, who was a *disinterested* person and one well known for his literary attainments.

H. H. Furness, Esq., an old friend of the late Dr. Constantine Hering, finally undertook the task. The profession remains under heavy obligations to this gentleman, who prepared himself for his task in diligently reading Hahnemann's and various other homœopathic authors' writings. Mr. Furness is not connected with the medical profession in any manner whatever; his labors are purely philanthropic. He was not aware, nor were Messrs. Hubbard, the publishers of the *Encyclopedia Americana*, that the consent and approval of the Committee of the American Institute were essentially necessary before said paper was published by them, and they are not aware of the fact that such consent or approval was *asked for* or was granted. On the face of the facts before us, the paper itself is credited to H. H. F., and *not* to H. H. F. representing the Committee of the American Institute.

The very fact that said paper emanated from a *disinterested* scholar, uninfluenced by professional parties, will give the paper more force and a greater acknowledgment than it would have received otherwise.

The Committee of the Institute were an *interested* party; Mr. Furness was, to all intents and purposes, a *disinterested* party, and his innate politeness and modesty would have debarred him from accepting any invitation to write said paper, nor would the Messrs. Hubbard have offered him one if either of them had been aware that the American Institute had appointed a committee to prepare said paper. Even after it was printed, it appears that said Committee had not then written such a paper, nor had they offered it for publication to the Messrs. Hubbard.

The facts are simply these: Mr. Furness wrote a paper on Homœopathy; that paper was published in the *Encyclopedia Americana,* and is copyrighted by the publishers, the Messrs. Hubbard. Further, that a copy of said paper was given the Committee of the American Institute of Homœopathy and was laid before the Institute, was approved by the Committee, was accepted and referred by the Institute, and a vote of thanks was extended to H. H. Furness, Esq., for the able manner in which he has treated *his* great subject.

*At last* the Institute has accepted as authentic the history, the development, and the fundamental principles governing the homœopathic healing art as they have now been laid down before the public. It is the first step toward abandoning a growing idea about the freedom of practice and the freedom assumed by homœopathic colleges to teach all sorts of principles of practice, as they may appear applicable under the guidance of individual judgment, however unhomœopathic they may appear to the strict Hahnemannian. *At last* we have the sanction of the American Institute of Homœopathy for a logical exposition of our noble healing art, and this great paper by Mr. Furness will do much to prevent reading and intelligent persons from falling into the hands of "pretenders." We also express the hope that the *International Hahnemannian Association* will extend a resolution of thanks to the philanthropic and able author of the above-mentioned paper at its next meeting.

